# Peripheral Blood S100B Levels in Autism Spectrum Disorder: A Systematic Review and Meta-Analysis

**DOI:** 10.1007/s10803-020-04710-1

**Published:** 2020-10-02

**Authors:** Zhen Zheng, Peng Zheng, Xiaobing Zou

**Affiliations:** 1 Department of Pediatrics, The Third Affiliated Hospital, Sun Yat-Sen University, Guangzhou, 510630 Guangdong China; 2 College of Horticulture, South China Agricultural University, Guangzhou, 510642 Guangdong China

**Keywords:** Autism spectrum disorder, Blood, S100B, Meta-analysis

## Abstract

The S100 calcium-binding protein beta subunit (S100B) protein, which mostly exists in the central nervous system, is commonly noted as a marker of neuronal damage. We conducted the first systematic review with meta-analysis to compare peripheral blood S100B levels in individuals with ASD with those in healthy controls. A systematic search was carried out for studies published before May 5, 2020. In total, this meta-analysis involved ten studies with 822 participants and 451 cases. The meta-analysis revealed that individuals with ASD had higher peripheral blood S100B levels than healthy controls [standardized mean difference (SMD) = 0.97, 95% confidence interval (95% CI) = 0.41–1.53; *p* < 0.001]. Peripheral blood S100B levels may have potential as a useful biomarker for ASD.

## Introduction

Autism spectrum disorder (ASD) comprises neurodevelopmental disorders specified by stereotypic behaviour, limited social interaction, and impaired communication. The worldwide prevalence of ASD is approximately 1% (Baxter et al. 2015). A higher prevalence of one per 54 children aged 8 years was reported in the United States, and the male-to-female ratio was about 4.3:1 (Maenner et al. 2020). The prevalence of ASD has increased rapidly, while there is a lack of specific treatment for ASD. ASD starts early and lasts throughout the lifetime, which affects the quality of life for individuals with ASD and places a financial burden on families. Therefore, ASD is considered a major concern for public health worldwide.

However, the cause of ASD has not yet been identified. Current evidence suggests that ASD may be a genetically and environmentally determined disorder (Mandy and Lai 2016; Robinson et al. 2016). To date, there are no specific biomarkers for ASD, and the diagnosis must be based on behaviour. Abnormal behaviour is not obvious until about 12–18 months old. In addition, ASD is correlated with mental diseases such as anxiety and schizophrenia (Lai et al. 2019; Zheng et al. 2018) but is not correlated with medical diseases such as allergies and asthma (Lyall et al. 2015; Zheng et al. 2016a). This complexity has spurred a search for biomarkers that can provide early diagnosis and predict treatment response. Multiple studies have reported that biomarkers like glutamate and brain-derived neurotrophic factor can help diagnose ASD and Alzheimer’s disease early (Qin et al. 2017, 2016; Zheng et al. 2016b, c). However, there is still a need for specific biomarkers relevant only to ASD.

The S100 calcium-binding protein beta subunit (S100B) protein is commonly noted as a marker of neuronal damage and blood–brain barrier dysfunction (Koh and Lee 2014). The S100B protein mainly exists in the Schwann cells and glial cells of the central nervous system. The S100B protein plays important roles in neuronal survival, differentiation, apoptosis, astrocytic proliferation and the regulation of neuroinflammation (Michetti et al. 2019). Evidence suggested that following inflammation and stress, neurons were damaged and blood–brain barrier was dysfunction. S100B may release from the damaged neurons into the bloodstream, and its concentration in the peripheral blood is increased (Michetti et al. 2019). Recently, important clinical findings indicated that peripheral blood S100B levels can rule out central nervous system damage which was measured by magnetic resonance imaging in patients (Moss et al. 2020; Thompson et al. 2016). Therefore, scientists assume that peripheral blood S100B levels may reflect the presence of neuropathological conditions (Hughes et al. 2018; Shotar et al. 2019). The measurement of S100B in peripheral blood is much easier than the direct evaluation of S100B in the brain, and S100B levels in peripheral blood may have potential as a useful biomarker for ASD.

The view that “the periphery is a window to the brain” prompted scientists to evaluate the correlation between peripheral blood S100B levels and ASD. But the correlation is still uncertain. Some studies have shown that individuals with ASD had higher peripheral blood S100B levels than healthy controls (Al-Ayadhi and Mostafa 2012; Guloksuz et al. 2017). Other studies have found that there is no correlation between peripheral blood S100B levels and ASD (Esnafoglu et al. 2017; Ma et al. 2019).

Thus, a meta-analysis on this topic is necessary. In this study, we systematically reviewed studies investigating the correlation between peripheral blood S100B levels and ASD. Then, subgroup and meta-regression analyses were carried out to estimate heterogeneity.

## Methods

### Literature Search

A search for relevant studies published before May 5, 2020 in the Embase, Scopus, PubMed, Cochrane Library, Web of Science, Wanfang, and China National Knowledge Infrastructure databases was performed. The terms applied for the database search included “autism spectrum disorder,” “ASD,” “autism,” “pervasive developmental disorder,” “Asperger syndrome,” “autistic disorder,” “S100B,” “S100 calcium-binding protein β subunit,” “S100b,” “S100β,” “S100B protein,” and “S100 calcium binding protein beta subunit.” Studies in humans and published in either the English language or the Chinese language were adopted.

### Study Selection

Inclusion criteria were (1) an evaluation of peripheral blood S100B levels in individuals with ASD and (2) a description of S100B levels in the median and range, the mean and standard deviation (SD), or the mean and standard error of the mean (SEM).

Exclusion criteria were (1) animal studies, reviews, abstracts, and letters; (2) study populations duplicated in another study; (3) studies that did not measure peripheral blood S100B levels, including pharmacology, genetics, brain imaging, and postmortem studies; and (4) studies that showed peripheral blood S100B levels in dot plot or histogram format, with no numerical results provided.

### Data Extraction

Data on the author, number of samples, number of males, participant age and publication year were extracted. Data on country, ASD diagnostic criteria, adjusted confounders, study design, analytical technology, biomaterial, S100B and measurement unit were also extracted. Different measurement units (ng/ml, pg/ml, or ug/l) were used in the studies. All S100B levels were reported in pg/ml in this meta-analysis. If data were expressed in terms of the median and range, the sample mean and SD were calculated according to the method of Wan et al. (Wan et al. 2014). If data were presented with the SEM, then the sample SD was calculated using the formula SD = SEM x √n (n: sample size).

### Quality Evaluation

The quality of the studies was estimated by the Newcastle–Ottawa Scale (NOS) (Stang 2010). The studies were evaluated as high (scored 7–9), medium (scored 4–6), or low (scored 0–3) quality.

### Statistical Analysis

The standard mean difference (SMD) was summarized to calculate peripheral blood S100B levels in ASD. Heterogeneity was determined by the I^2^ and Q statistics. The I^2^ values of 0.25, 0.5, and 0.75 revealed small, medium and high heterogeneity, respectively.

Publication bias was confirmed by funnel plot analysis as well as by Begg’s and Egger’s tests. Subgroup analyses were carried out by using study design, biomaterial, analytical technology, and geographic location. Meta-regressions were conducted to evaluate whether sample size, mean age, publication year, and gender affected the outcome. Sensitivity analysis was carried out by the omission of studies one by one to evaluate whether any study changed the overall outcome. Stata 12.0 and Review Manager 5.1.2 software were used to perform the statistical tests.

## Results

### Literature Search

**Fig. 1:**
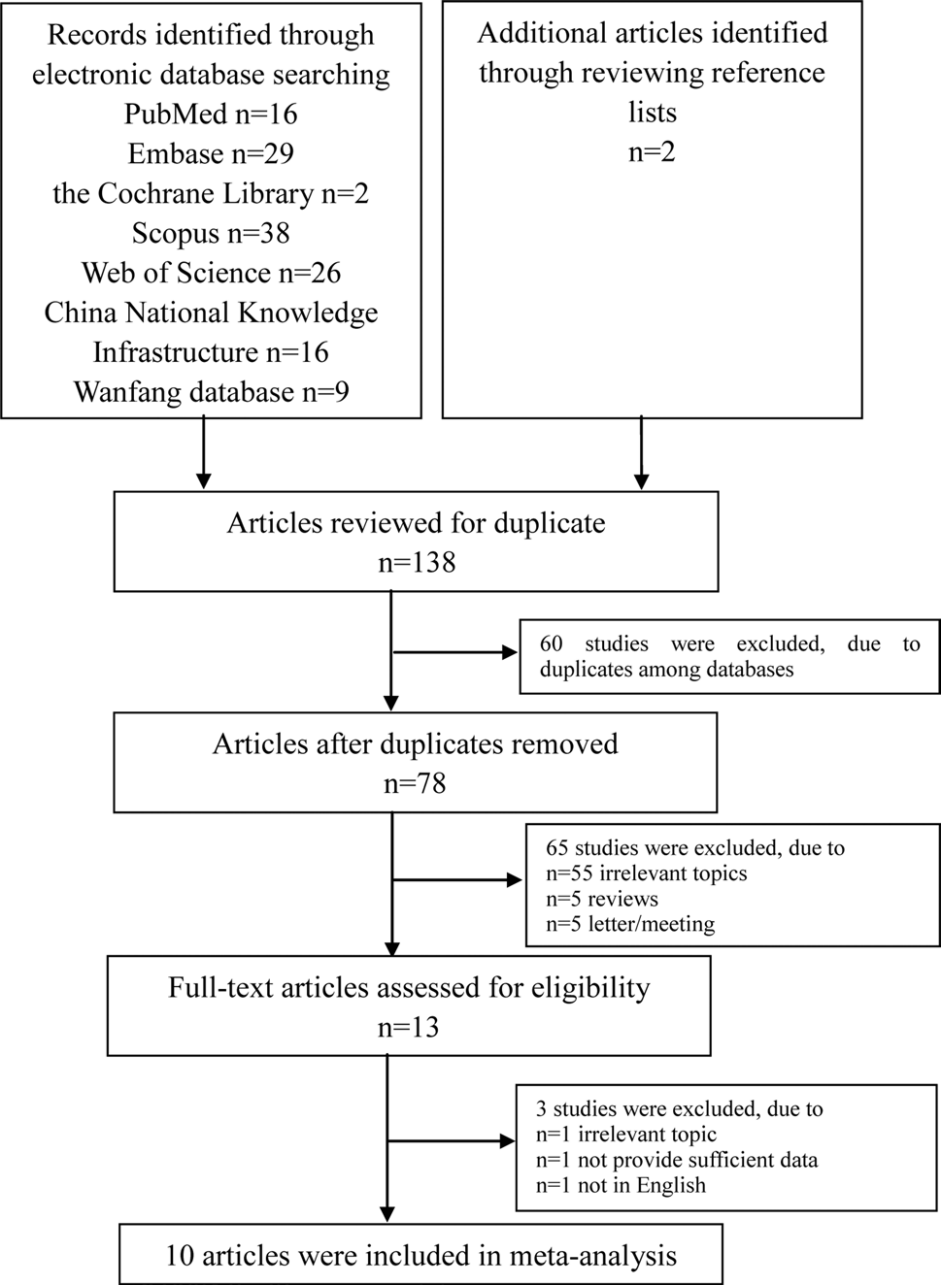
Flow-chart of study selection

We first conducted a systematic search and identified 16 studies from PubMed, 29 studies from Embase, two studies from the Cochrane Library, 38 studies from Scopus, 26 studies from the Web of Science, 16 studies from China National Knowledge Infrastructure, nine studies from the Wanfang database, and two additional studies from the scanning of references. After reviewing titles and abstracts, 13 papers on peripheral blood S100B levels in ASD were selected for full-text review. Subsequently, one study was excluded due to its irrelevant topic. One report without sufficient data was excluded. One report was excluded for not being published in English. Eventually, 10 studies with 822 participants and 451 cases met the criteria for inclusion in the present meta-analysis (Fig. 1).

### Study Characteristics

The included studies were published from 2012 to 2019 (Abou-Donia et al. 2019; Al-Ayadhi and Mostafa 2012; Ayaydin and Kirmit 2020; Esnafoglu et al. 2017; Guloksuz et al. 2017; Ma et al. 2019; Peng and Zhu 2017; Shaker et al. 2016; Tomova et al. 2019; Zhou 2015) (Table 1). Ten studies encompassed 822 participants and 451 cases. Three studies were performed in China (Ma et al. 2019; Peng and Zhu 2017; Zhou 2015), three in Turkey (Ayaydin and Kirmit 2020; Esnafoglu et al. 2017; Guloksuz et al. 2017), one in the USA (Abou-Donia et al. 2019), one in Saudi Arabia (Al-Ayadhi and Mostafa 2012), one in Egypt (Shaker et al. 2016), and one in the Slovak Republic (Tomova et al. 2019). The sample sizes ranged from 10 (Abou-Donia et al. 2019) to 89 (Tomova et al. 2019). The mean age of participants varied from 1.8 (Peng and Zhu 2017) to 18 (Abou-Donia et al. 2019) years old. The mean S100B values of cases ranged from 0.36 ± 0.19 pg/ml (Abou-Donia et al. 2019) to 298,630 ± 161,140 pg/ml (Ma et al. 2019). Two different biomaterials for S100B assays were used in this systematic review: serum (Abou-Donia et al. 2019; Al-Ayadhi and Mostafa 2012; Ayaydin and Kirmit 2020; Esnafoglu et al. 2017; Ma et al. 2019; Peng and Zhu 2017; Shake 2016; Zhou 2015) and plasma (Guloksuz et al. 2017; Tomova et al. 2019). Moreover, 9 studies performed S100B assessment using enzyme linked immunosorbent assay (ELISA) (Al-Ayadhi and Mostafa 2012; Ayaydin and Kirmit 2020; Esnafoglu et al. 2017; Guloksuz et al. 2017; Ma et al. 2019; Peng and Zhu 2017; Shaker et al. 2016; Tomova et al. 2019; Zhou 2015) as an analytical procedure, whereas 1 adopted Western blot (WB) (Abou-Donia et al. 2019). In addition, ten studies considered gender and age as adjusted confounders (Abou-Donia et al. 2019; Al-Ayadhi and Mostafa 2012; Ayaydin and Kirmit 2020; Esnafoglu et al. 2017; Guloksuz et al. 2017; Ma et al. 2019; Peng and Zhu 2017; Shaker et al. 2016; Tomova et al. 2019; Zhou 2015). Two studies considered body mass index (BMI) as adjusted confounder (Esnafoglu et al, 2017; Guloksuz et al, 2017). One study considered socioeconomic status as adjusted confounder (Shaker et al. 2016).

**Table 1:** Characteristics of the studies

First author, year	Country	Study design	Sample sizes ASD/controls	Male (n) ASD/controls	Age Mean ± SD(range) ASD/controls	ASD diagnostic criteria	Analytical technology	Biomaterial	S100B Mean ± SD ASD/controls	Unit of measure	Adjusted confounders
Abou-Donia MB,2019	USA	Cross-sectional	10/10	2/2	3–18 6–12	ADIR、ADOS、DSM-V	WB	Serum	0.36 ± 0.19 0.20 ± 0.13	None	Gender, age
Al-Ayadhi LY,2012	Saudi Arabia	Cross-sectional	64/46	50/34	8.4 ± 2.5 9.1 ± 2.4	DSM-IV	ELISA	Serum	207.97 ± 52.6 171.33 ± 34.65	pg/ml	Gender, age
Ayaydin H,2020	Turkey	Cross-sectional	43/41	30/31	6 ± 2.2 7 ± 2.2	DSM-IV	ELISA	Serum	49.13 ± 1.57 24.33 ± 24.2	pg/ml	Gender, age
Esnafoglu E,2017	Turkey	Cross-sectional	35/31	26/25	7.6 ± 3.62 6.85 ± 3.16	DSM-V	ELISA	Serum	3.33 ± 4.19 3.42 ± 4.56	pg/ml	Gender, age, BMI
Guloksuz SA,2017	Turkey	Cross-sectional	40/35	30/22	7.13 ± 3.89 6.75 ± 3.96	DSM-IV	ELISA	Plasma	182.50 ± 34.61 48.13 ± 13.17	pg/ml	Gender, age, BMI
Ma F,2019	China	Cross-sectional	35/40	22/25	4.3 ± 0.5 4.4 ± 0.6	DSM-V	ELISA	Serum	298,630 ± 161,140 272,440 ± 122,280	pg/ml	Gender, age
Peng ZQ,2017	China	Cross-sectional	65/72	53/61	1.8–9.5 2.1–9.4	DSM-IV	ELISA	Serum	296,700 ± 154,690 244,390 ± 154,060	pg/ml	Gender, age
Shaker NM,2016	Egypt	Case–control	30/22	27/20	3–14 3–13	ICD-10	ELISA	Serum	123.8 ± 123.4 112.8 ± 9.3	pg/ml	Gender, age, socioeconomic status
Tomova A,2019	Slovak Republic	Cross-sectional	89/34	89/34	2–16 2–12	DSM-V、ADOS-2、ADIR	ELISA	Plasma	33.04 ± 31.6 23.26 ± 16.7	pg/ml	Gender, age
Zhou YY,2015	China	Cross-sectional	40/40	32/30	2–5 2–5	ABC	ELISA	Serum	195.43 ± 41.62 151.88 ± 21.06	pg/ml	Gender, age

To identify ASD cases, researchers adopted the diagnostic criteria from the Autism Diagnostic Interview-Revised (ADI-R) (Abou-Donia et al. 2019; Tomova et al. 2019), Diagnostic and Statistical Manual (DSM-IV) (Al-Ayadhi and Mostafa 2012; Ayaydin and Kirmit 2020; Guloksuz et al. 2017; Peng and Zhu 2017), DSM-V (Abou-Donia et al. 2019; Esnafoglu et al. 2017; Ma et al. 2019; Tomova et al. 2019), Autism Diagnostic Observation Schedule (ADOS) (Abou-Donia et al. 2019), ADOS-2 (Tomova et al. 2019), Autism Behavior Checklist (ABC) (Zhou 2015), and International Classification of Diseases, 10th Revision (ICD-10) (Shaker et al. 2016).

### Main Correlation of Peripheral Blood S100B Levels with ASD

**Fig. 2:**
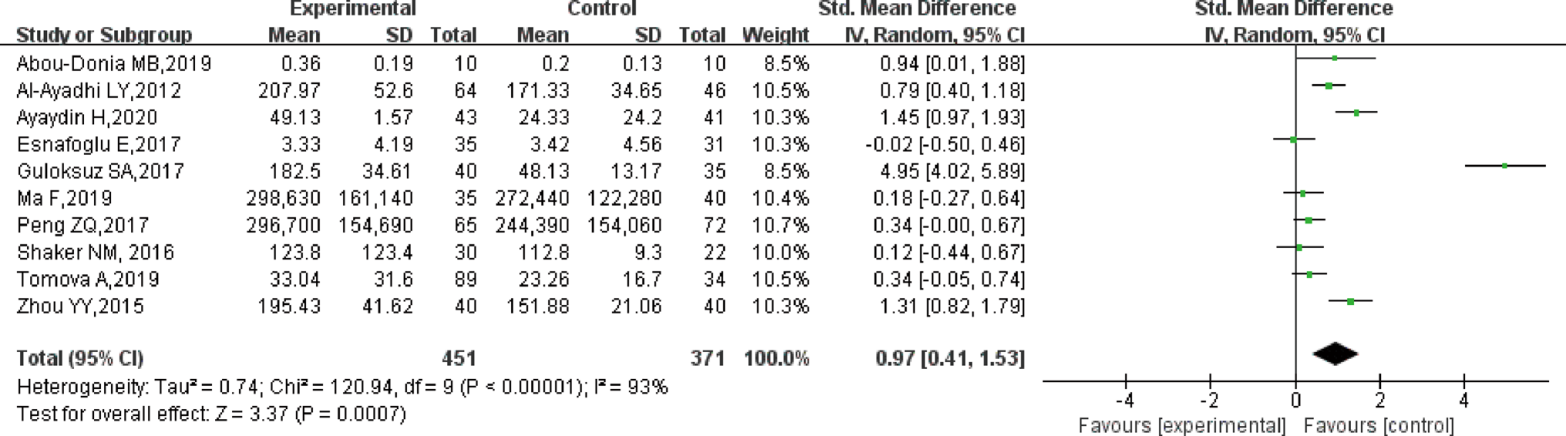
Forest plot of the relative peripheral blood S100B levels in individuals with ASD

The results showed that individuals with ASD had higher peripheral blood S100B levels than healthy controls [SMD = 0.97, 95% confidence interval (95% CI) = 0.41–1.53; *p* < 0.001] but with significant heterogeneity (I^2^ = 93%,* p* < 0.001) (Fig. 2).

### Quality Evaluation

The quality evaluation results showed that the ten studies were high quality. The mean score was 7.7 (Table 2).

**Table 2:** Quality assessment of the studies

First author, year	Study design	Selection	Comparability	Exposure/Outcome	Total scores
Abou-Donia et al. 2019	Cross-sectional	★★★★	★★	★★★	9
Al-Ayadhi and Mostafa 2012	Cross-sectional	★★★★	★★	★★	8
Ayaydin and Kirmit 2020	Cross-sectional	★★★	★★	★★	7
Esnafoglu et al. 2017	Cross-sectional	★★★	★★	★★★	8
Guloksuz et al. 2017	Cross-sectional	★★★	★★	★★	7
Ma et al. 2019	Cross-sectional	★★★★	★★	★★	8
Peng and Zhu 2017	Cross-sectional	★★★★	★★	★★	8
Shaker 2016	Case–control	★★★	★★	★★	7
Tomova et al. 2019	Cross-sectional	★★★★	★★	★★	8
Zhou 2015	Cross-sectional	★★★	★★	★★	7

### Publication Bias

**Fig. 3:**
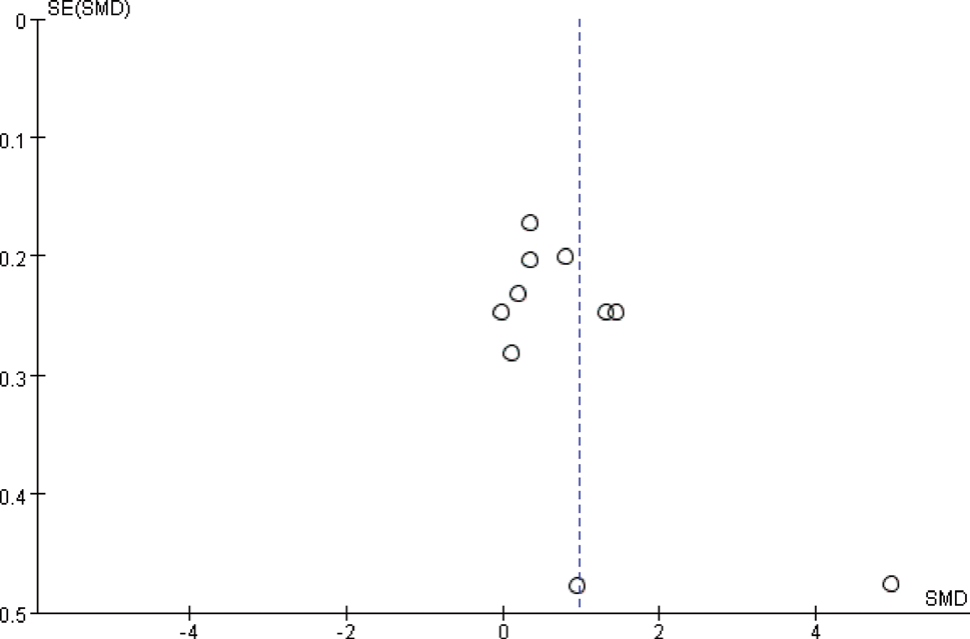
Funnel plot of the relative peripheral blood S100B levels in individuals with ASD

Asymmetry was found by funnel plot analysis (Fig. 3). Begg’s and Egger’s tests demonstrated no obvious publication bias (Begg’s test, *p* = 0.37; Egger’s test, *p* = 0.07).

### Subgroup Analysis

We performed subgroup analysis based on study design. The pooled SMDs were 1.07 (95% CI = 0.46–1.68, *p* < 0.001) for cross-sectional studies and 0.12 (95% CI = −0.44–0.67,* p* = 0.68) for case–control study. Further subgroup analysis of biomaterials showed that individuals with ASD had higher peripheral blood S100B levels in serum [0.62 (95% CI = 0.24–1.01, *p* = 0.002)] but not in plasma [2.63 (95% CI = −1.89–7.15,* p* = 0.25)]. In addition, we conducted subgroup analysis on the basis of which analysis techniques were applied. The effect values of S100B levels in individuals with ASD were 0.94 (95% CI = 0.01–1.88, *p* = 0.05) for WB and 0.97 (95% CI = 0.37–1.57, *p* = 0.001) for ELISA. We also carried out subgroup analysis in terms of geographic locations. The results demonstrated that individuals with ASD had higher peripheral blood S100B levels in other countries [0.5 (95% CI = 0.16–0.85, *p* = 0.004)] but not in China [0.6 (95% CI =−0.03–1.23, *p* = 0.06)] or Turkey [2.09 (95% CI = −0.16–4.34, *p* = 0.07)] (Table 3).

**Table 3:** Stratified analysis of peripheral blood S100B levels in individuals with ASD

Variables	No. of comparisions	No. of subjects		Meta-analysis				Heterogeneity		Test for subgroup differences	
		ASD	Controls	SMD	95%CI		*p*-value	I^2^	*p*-value	I^2^	*p*-value
Study design											
Case–control	1	30	22	0.12	−0.44	0.67	0.68	Not applicable	Not applicable	80.6	0.02
Cross-sectional	9	421	349	1.07	0.46	1.68	< 0.001	93	< 0.001		
Biomaterial											
Plasma	2	129	69	2.63	−1.89	7.15	0.25	99	< 0.001	0	0.39
Serum	8	322	302	0.62	0.24	1.01	0.002	81	< 0.001		
Analytical technology											
WB	1	10	10	0.94	0.01	1.88	0.05	Not applicable	Not applicable	0	0.95
ELISA	9	441	361	0.97	0.37	1.57	0.001	93	< 0.001		
Geographic location											
China	3	140	152	0.6	−0.03	1.23	0.06	85	0.001	0	0.39
Turkey	3	118	107	2.09	−0.16	4.34	0.07	98	< 0.001		
Others	4	193	112	0.5	0.16	0.85	0.004	45	0.14		

*No*. number, *CI* confidence interval.

### Meta-Regression Analysis

The meta-regression analyses indicated that none of the covariates (sample size, mean age, publication year, and gender) significantly influenced the results (Table 4).

**Table 4:** Meta-regression of peripheral blood S100B levels in individuals with ASD

Moderator	No. of comparisions	No. of subjects		Meta-regression				Proportion of variance explained
		ASD	Controls	Slope	95%CI		*p*-value	R^2^ analog
Age(mean, years)	10	451	371	0.77	−1.58	3.15	0.47	−5.76
Gender(%man)	10	451	371	−0.52	−0.07	0.04	0.62	−9.38
Sample size	10	451	371	−0.3	−0.04	0.029	0.77	−12.83
Publication year	10	451	371	−0.03	−0.5	0.49	0.98	−14.66

*No*. number, *CI* confidence interval.

### Sensitivity Analysis

Sensitivity analysis revealed that the overall results were not affected by a particular study.

## Discussion

To our knowledge, this is the first meta-analysis to explore peripheral blood S100B levels in ASD. Pooling the ten studies, 822 participants and 451 cases were encompassed. Our study showed that individuals with ASD had higher peripheral blood S100B levels than healthy controls. Sensitivity analysis indicated that the outcome was not unduly influenced by a particular study. In addition, no obvious publication bias was observed. This study demonstrated that individuals with ASD had higher peripheral blood S100B levels.

High levels of heterogeneity were detected in this study. Subgroup analyses revealed that different study designs contributed to the heterogeneity. Individuals with ASD who had higher peripheral blood S100B levels were detected in cross-sectional studies but not in case–control study, indicating that the association may be affected by the study design. But this analysis may have been underpowered owing to only one case–control study was included in the subgroup. This potential confounding factor needs to be considered in the future analysis of S100B in individuals with ASD.

Individuals with ASD had higher S100B levels when measured in serum but not when measured in plasma. In the meta-analysis, most studies provided serum S100B levels; therefore, enough data were available to conclude that ASD is correlated with elevated serum S100B levels. Evidence that changes in serum S100B levels may reflect changes occurring in the brain has been reported (Vos et al. 2010). In some central nervous system disorders, such as intracerebral haemorrhage and ischaemia–reperfusion injury, the increase in serum S100B levels was consistently detected by biochemical analyses of S100B levels in the brain (Liu et al. 2015; Neves et al. 2017). Furthermore, serum S100B levels have been used as a standard indicator of treatment response in cerebral ischaemia–reperfusion injury (Li and Liu 2017). Therefore, in our meta-analysis, elevated serum S100B levels in individuals with ASD may reflect what is happening in the brains of individuals with ASD. Few studies have analyzed plasma S100B levels. It is necessary to further study the plasma S100B levels in this population.

Interestingly, the peripheral blood S100B levels were higher in individuals with ASD than in healthy controls when measured by ELISA but not when measured by WB, indicating that different assay methods may affect the correlation. Assay properties such as sensitivity and inter-assay and intra-assay coefficients of variability should be considered. In addition, differences in the way samples are collected and handled might contribute to the discrepancies in reported S100B levels in individuals with ASD. But only one study utilized WB in the subgroup, the result was not robust. Further investigations are needed to explore the correlation between S100B levels measured by WB and ASD.

In addition, individuals with ASD were discovered to have higher peripheral blood S100B levels than healthy controls in other countries, but there was no difference between the groups in China or Turkey. Genetic factors, environmental factors, lifestyle and economic conditions may contribute to this difference.

Furthermore, some clinical confounders were not analyzed in our meta-analysis, such as BMI, leptin and other adipose-related factors, may contribute to the observed heterogeneity. Scientists have reported that S100B levels were closely correlated with BMI, leptin and other adipose-related factors (Steiner et al. 2010). The studies included in our meta-analysis did not report BMI, leptin and other adipose-related factors, which made it impossible to analyze whether they were confounders contributed to the heterogeneity. It raises awareness of the need to consider these factors in future works.

The mechanisms of increased S100B in ASD are not completely clear, but several mechanisms have been proposed. First, autoimmunity may be involved in the development of ASD (Edmiston et al. 2017). Brain-specific autoantibodies have been found in individuals with ASD (Ramirez-Celis et al. 2020). Autoantibodies can bind to brain tissue antigens by passing through the blood–brain barrier, forming immune complexes and leading to neuronal damage (Al-Ayadhi and Mostafa 2012). Elevated S100B levels may indicate neuronal damage in ASD. Second, neuroinflammation was found in the brains of individuals with ASD (Matta et al. 2019). It has been reported that S100B may modulate cytokine secretion and may also be modulated by pro-inflammatory cytokines (Di Sante et al. 2020). S100B may function as a cytokine and support the implication of neuroinflammation in the development of ASD (Shaker NM 2016). Third, S100B has both trophic and toxic effects on neurons, depending on its concentration. When S100B levels are excessively increased, S100B acts as a neurotoxic protein that induces apoptosis. Ayaydın H et al. (Ayaydin and Kirmit 2020) reported that S100B plays an important role in neuronal apoptosis, which may contribute to the pathogenesis of ASD. The pathophysiological roles of S100B in ASD are not well explained, and more studies are warranted to verify this assumption in the future.

This meta-analysis had the following strengths. First, this is the first meta-analysis carried out to evaluate peripheral blood S100B levels in ASD. Second, no significant publication bias existed, indicating the high reliability of the results. Third, the sensitivity analysis found that no study could change the outcome, demonstrating that the conclusion was robust.

However, there were some limitations. First, because of the small sample size, studies with larger samples are required to illustrate the role of peripheral blood S100B levels in ASD. Second, since only a few studies analyzed the severity of ASD and S100B levels, the correlation between them was not evaluated. Future studies should pay attention to this subject. Third, the meta-analysis provides us with comprehensive results mainly from cross-sectional studies. Therefore, whether S100B levels are the cause or the result of ASD development remains unclear. Fourth, significant heterogeneity was found in this meta-analysis. Random-effects, subgroup and regression analyses were performed, and different study designs explained the heterogeneity. But residual confounding factors such as a wide range of mean S100B values reported across studies, which may be related with the different measurement units and different analytical technologies used in the included studies, are still a problem worthy of attention. Therefore, heterogeneity may influence the accuracy of the final results.

Despite these limitations, this study may have clinical application value for using S100B levels in the early identification and evaluation of ASD progression. This meta-analysis provides clinical evidence that individuals with ASD have increased peripheral blood S100B levels. Scientists have reported that changes in peripheral blood S100B levels may reflect changes occurring in the brain (Vos et al. 2010). Thus, higher peripheral blood S100B levels may reveal higher brain S100B levels in individuals with ASD. Brain S100B levels in individuals with ASD are warranted to be evaluated to confirm this hypothesis, which may further support that S100B can be used as a reliable biomarker for ASD.

## Conclusions

This analysis provides evidence that individuals with ASD have increased peripheral blood S100B levels. Peripheral blood S100B levels may have potential as a useful biomarker for ASD. Studies with large samples are warranted to investigate the severity of the clinical symptoms and the causal relationship between ASD and S100B levels.
